# Recent Loss of Vitamin C Biosynthesis Ability in Bats

**DOI:** 10.1371/journal.pone.0027114

**Published:** 2011-11-01

**Authors:** Jie Cui, Xinpu Yuan, Lina Wang, Gareth Jones, Shuyi Zhang

**Affiliations:** 1 Institute of Molecular Ecology and Evolution, Institutes for Advanced Interdisciplinary Research, East China Normal University, Shanghai, China; 2 School of Biological Sciences, University of Bristol, Bristol, United Kingdom; University of Western Ontario, Canada

## Abstract

The traditional assumption that bats cannot synthesize vitamin C (Vc) has been challenged recently. We have previously shown that two Old World bat species (*Rousettus leschenaultii* and *Hipposideros armiger*) have functional L-gulonolactone oxidase (GULO), an enzyme that catalyzes the last step of Vc biosynthesis *de novo*. Given the uncertainties surrounding when and how bats lost *GULO* function, exploration of gene evolutionary patterns is needed. We therefore sequenced *GULO* genes from 16 bat species in 5 families, aiming to establish their evolutionary histories. In five cases we identified pseudogenes for the first time, including two cases in the genus *Pteropus* (*P. pumilus* and *P. conspicillatus*) and three in family Hipposideridae (*Coelops frithi*, *Hipposideros speoris*, and *H. bicolor*). Evolutionary analysis shows that the *Pteropus* clade has the highest ω ratio and has been subjected to relaxed selection for less than 3 million years. Purifying selection acting on the pseudogenized *GULO* genes of roundleaf bats (family Hipposideridae) suggests they have lost the ability to synthesize Vc recently. Limited mutations in the reconstructed *GULO* sequence of the ancestor of all bats contrasts with the many mutations in the ancestral sequence of recently emerged *Pteropus* bats. We identified at least five mutational steps that were then related to clade origination times. Together, our results suggest that bats lost the ability to biosynthesize vitamin C recently by exhibiting stepwise mutation patterns during *GULO* evolution that can ultimately lead to pseudogenization.

## Introduction

Vitamin C (Vc), or L-ascorbic acid is a water-soluble vitamin that is an essential nutrient impotant in animal metabolism. Vc is involved in tissue growth and repair, and also functions as an antioxidant to block damage caused by free radicals. It is also a cofactor in enzymatic reactions that are catalyzed by Cu^+^-dependent monooxygenases and Fe^2+^-dependent dioxygenases [1]. Vc is required in the diet of all vertebrates in order to sustain good health [2], and Vc deficiency can lead to potentially fatal scurvy in humans. Most vertebrates can satisfy their Vc requirements by synthesizing it *de novo* with glucose [3]. However, some mammals, including haplorhine primates and guinea pigs, have lost this ability, and thus have to obtain Vc from their diet [4]. The ability to synthesize Vc has been reported in many ancestral vertebrate lineages [5], [6], suggesting the ability for *de novo* synthesis is ancient. Moreover, there is an apparent transition of the organs used for the biosynthesis of Vc during evolution, from the kidney of reptiles to the liver of mammals [7].

The ability to synthesize Vc has been lost independently several times in vertebrates e.g. in some fishes [5], in some passeriform birds [7], in some bats [8], in guinea pigs [10] and in primates of the suborder Haplorrhini (e.g. monkeys, apes and humans) [7], [9]–[10]. All of these species lack activity of L-Gulonolactone oxidase (GULO) in their livers (or kidneys) to catalyze the last step of the Vc synthesis pathway so that they need to compensate by obtaining Vc from their food [8]–[10]. The gene encoding GULO in guinea pigs and humans has become a pseudogene [11], [12].

Our recent research has challenged the traditional opinion that bats cannot synthesize Vc [8], [13] by showing that *GULO* genes in two species (*Rousettus leschenaultii* and *Hipposideros armiger*) are still in their intact forms and can produce functional proteins [14]. Bats are perhaps in the process of large-scale loss of Vc biosynthesis ability [14], and show varying degrees of lack of GULO function. For example, the genera *Pteropus* and *Rousettus* belong to the same chiropteran family (Pteropodidae), and although the former has lost the ability to synthesize Vc, the latter retains it [14].

Our previous study on Vc synthesis in bats raises the question-what is the evolutionary pattern that shapes bat *GULO* evolution in bats? Given the uncertainty of when and how bats lost *GULO* gene function, it is important to sequence *GULO* genes of more bat to explore patterns of *GULO* evolution. In this study, we therefore sequenced the *GULO* genes of 16 bat species and aimed to reconstruct bat *GULO* evolutionary history. Using ancestral reconstructions, we infer stepwise mutation patterns showing how bats may have lost *GULO* function.

## Materials and Methods

### Bat taxonomic coverage

Bat wing membrane biopsy specimens were taken from a collection at the Institute of Molecular Ecology and Evolution, East China Normal University. All experiments were conducted under permission to use these specimens granted by the by Animal Care Ethics Committee, East China Normal University (approval ID 20091225). Our screening included 16 bat species from 5 families: Pteropodidae, Hipposideridae, Rhinolophidae, Megadermatidae, and Rhinopomatidae, and sampling locations include China, Cameroon, Australia, India, and Vietnam (supplementary table S1).

### Bat *GULO* cloning and sequencing

Genomic DNA was extracted by using DNeasy Blood and Tissue Kit (Qiagen) according to the manufacturer's protocols. We aimed to amplify exon-3 to exon-8, the six exons encoding the functional region of the GULO enzyme (there are 12 exons in the gene in total), of 16 bat species (supplementary table S1) by using a series of primer pairs (supplementary table S2) designed according to the genomic sequences of *P. vampyrus* (GeneScaffold_1205), dog (*Canis familiaris*, ENSCAFT00000013370), cow (*Bos taurus*, ENSBTAT00000052052) and pig (*Sus scrofa*, ENSSSCT00000010600) that contained *GULO* genes from the Ensembl database (http://www.ensembl.org/). Polymerase chain reactions (PCR) were performed using Ex Taq^TM^ polymerase (TaKaRa) with the reaction conditions as follows: 94°C for 5 min followed by 30 cycles consisting of 94°C for 30 sec, 57–62°C for 15–30 sec, 72°C for 1 min, and a final extension of 72°C for 10 min. All PCR products were ligated into pGEM-T Easy vectors (Promega) and transformed. The universal T7 (5′-TAA TAC GAC TCA CTA TAG GG-3′) and SP6 (5′-ATT TAG GTG ACA CTA TAG-3′) sequencing primers were used to sequence all positive clones on an ABI 3730 DNA sequencer (Applied Biosystems). All new sequences are submitted to GenBank (supplementary table S1).

### Phylogenetic construction

To reconstruct the bat *GULO* phylogeny, we first retrieved non-bat orthologous genes in GenBank (www.ncbi.nlm.nih.gov/genbank/): horse ( *Equus caballus*, XM_001492727), dog (*Canis familiaris*, XM_543226), pig (*Sus scrofa*, NM_001129948), cow (*Bos taurus*, NM_001034043), panda (*Ailuropoda melanoleuca*, XM_002914414), rabbit (*Oryctolagus cuniculus*, XM_002709304), rat (*Rattus norvegicus*, NM_022220), mouse (*Mus musculus*, NM_178747), opossum (*Monodelphis domestica*, XM_001380006) and platypus (*Ornithorhynchus anatinus*, XM_001521551). We then aligned all these sequences with the bat sequences using ClustalW [15] implemented in MEGA4 [16]. Indels (deletions or insertions) and premature stop codons were excluded from the sequences before alignment. Because *GULO* genes are highly conserved in most mammals, evolutionary history was inferred using the Neighbor-joining (NJ) method, which was perfectly used in many studies [17]. All nucleotide positions were included. The evolutionary distances were computed using the Maximum Composite Likelihood model [18] with branch lengths represent genetic distances. All positions containing gaps and missing data were eliminated. Bootstraping with 2,000 replicates [19] was used to test phylogenetic robustness and nodes with bootstrap values lower than 50% were collapsed. Evolutionary analyses were conducted in MEGA4 [16].

### Evolutionary analyses

A widespread method used to inter selection pressures acting on specific genes is to estimate non-synonymous (dN) and synonymous (dS) nucleotide substitution rates and their ratio dN/dS (ω) [20]. We used the CODEML program with the likelihood method implemented in PAML4.4 [20] to evaluate selection pressures acting on each lineage of bat *GULO* genes mapped onto the published species tree [21], [22]. Two models were employed: the free-ratio model that allows the ω ratios to vary for each branch, and the two-ratio model that compares two different ω ratios between specified branches (e.g. *Pteropus* bats) and other branches. To test for the significance of each model used, likelihood ratio tests (LRTs) [23] were implemented, conducted by comparing twice the difference in likelihood between nested statistical models, i.e. the free-ratio versus the one-ratio model (which assumes an average ω for all lineages), and we also compared two-ratio versus one-ratio models.

To trace the amino acid changes during *GULO* gene evolution, ancestral reconstruction was employed [24]. The program CODEML using the empirical Bayes method in PAML4 [20], [24] was used for reconstructing amino acids in extinct ancestors on the species tree [21], [22]. Different amino acid changes were recorded after alignment using reconstructed ancestral sequences.

## Results

### Bat *GULO* cloning

**Figure 1:**
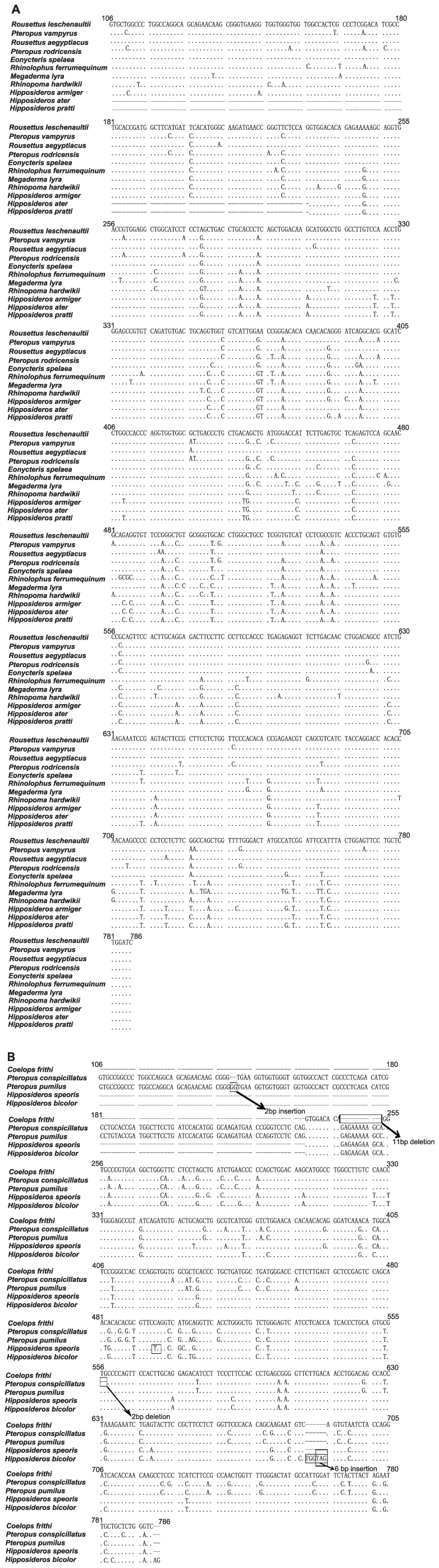
Alignments of bat *GULO* nucleotide gene sequences. (A) The intact bat *GULO* nucleotide gene sequences; (B) the bat *GULO* pseudogenized nucleotide gene sequences. The nucleotide position numbers are denoted according to the nucleotide sequence of *Rousettus leschenaultii* GULO (HQ415789). The boxes donate insertions, deletions, or premature stop codons that break the gene reading frames.

**Figure 2:**
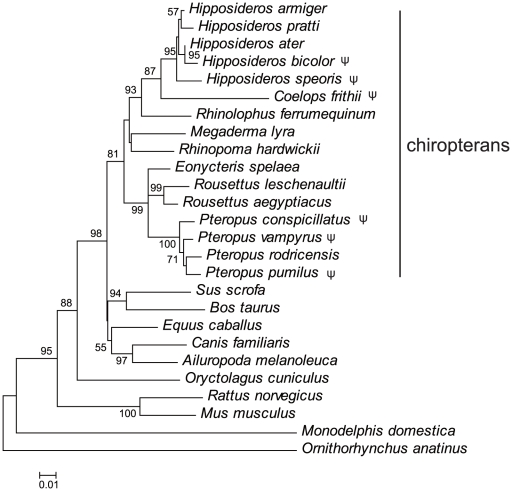
Phylogenetic tree based on *GULO* genes. The evolutionary history was reconstructed using the NJ method in MEGA4 [16]. The bootstrap consensus tree inferred from 2,000 replicates is shown to represent *GULO* evolution for each taxon. Bootstrap values lower than 50% are no shown. The scale bar represents genetic distance. The evolutionary distances were computed using the Maximum Composite Likelihood model. Lineages for pseudogenes are marked with ψ.

Bats showed lineage-specific gene pseudogenization including premature stop codons, insertions and deletions. Basically, our molecular cloning revealed two major patterns: 1) an intact *GULO* form: *Rousettus leschenaultii*, *R. aegyptiacus*, *Pteropus rodricensis*, *P. vampyrus*, *Eonycteris spelaea* (Pteropodidae), *Rhinolophus ferrumequinum*, *Hipposideros armiger*, *H. ater*, *H. pratti* (Hipposideridae), *Megaderma lyra* (Megadermatidae), and *Rhinopoma hardwickii* (Rhinopomatidae) (figure 1A); 2) pseudogenized form: *Pteropus conspicillatus*, *P. pumilus* (Pteropodidae), *Coelops frithii*, *Hipposideros bicolor*, and *H. speoris* (Hipposideridae) (figure 1B).

### Patterns of selection in bat *GULO* gene evolution

**Figure 3:**
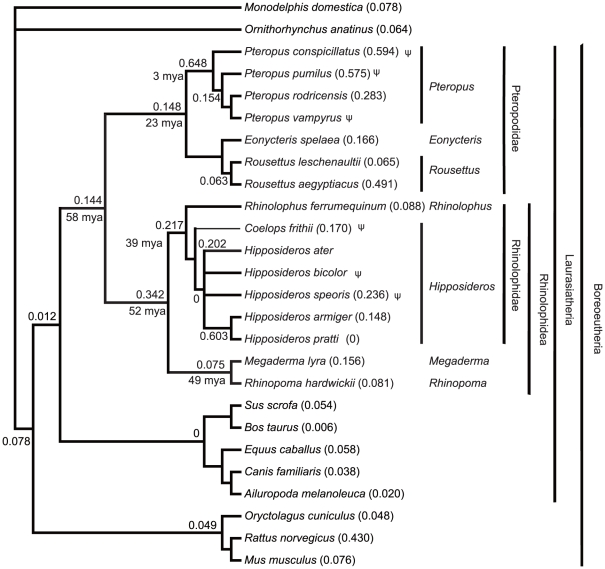
Selection pressures acting on *GULO* genes. The published species tree [22], [23] is shown and selection pressures marked were calculated using the free-ratio model in PAML4 [20]. Values given on the branches, or in parentheses, are ω ratios (dN/dS) estimated by maximum-likelihood. Values of infinity (∞, dS  = 0) are not shown. The time of origin for each ancestral node was collected from published data [22], [24]. Lineages for pseudogenes are marked with ψ.

The phylogenetic gene tree for *GULO* (figure 2) closely resembled the published species tree based on large-scale gene sequencing [21], [22]. Our selection tests showed high ω ratios (*P*-value  = 5.0×10^−6^, LRT) in all main clades of bats, giving a 12–54x higher ω ratio than in the ancestor of Laurasiatheria species (figure 3). The *Pteropus* clade has the highest ω ratio of 0.648. A pair of two-ratio models (with the *Pteropus* clade was set as the foreground) was then constructed by having the ω ratio fixed to 1 (neutral evolution) in one model and a ω ratio not fixing the other. LRT showed no significance when comparing the two models (*P*-value  = 0.677), which suggests that the evolution of *Pteropus* clade *GULO* genes is close to being neutral.

The higher ω ratios of *Pteropus* suggest their *GULO* genes have already been subjected to relaxed selection (over a period of less than 3 million years ago (mya), the origination time of this clade) [21], [22]. *GULO* genes of *Coelops frithii*, *Hipposideros armiger*, and *H. pratti* may have been at the early stages of pseudogenization because these genes have relatively low ω ratios (suggesting they are still under purifying selection). Several two-ratio models were also established with different bat clades as the foreground. LRTs (two-ratio model versus one-ratio model) showed significance only in the *Pteropus* clade (data not shown), supporting the above conclusion that this clade has subjected to relaxed selection.

### Ancestral reconstruction reveals stepwise mutation patterns

**Figure 4:**
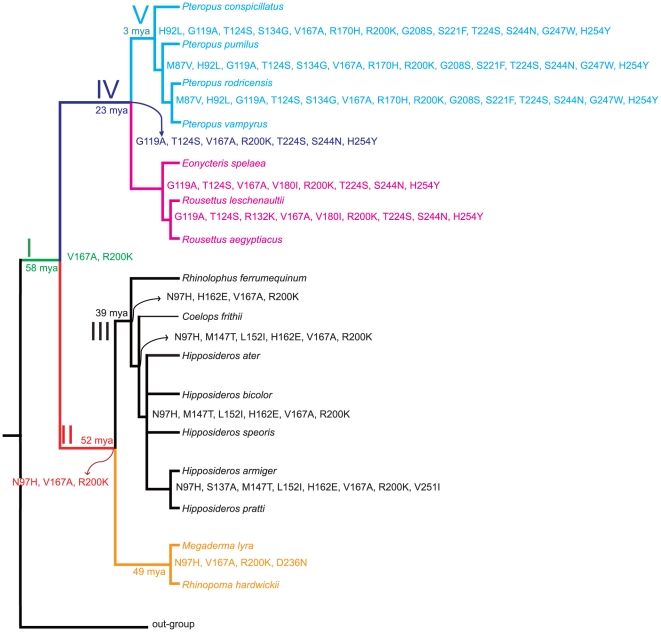
Stepwise mutation patterns during bat *GULO* evolution. Amino acid (abbreviation) changes of bat *GULO* are shown after the divergence of bats with non-bat Laurasiatheria species. The topology was retrieved from published species trees [21], [22]. Five major steps were identified according to the mutation pattern, and they are highlighted in Roman characters (I, II, III, IV, and V). The time of origin for each node was collected from published data [21], [25]. The positions of mutations are recorded according to the protein sequence of the *Rousettus leschenaultii* GULO gene (ADP88813). For example, G119A means that the amino acid G evolved from A at amino acid position 119. The outgroup of non-bat Laurasiatheria species include pig, cow, horse, cat, and panda.

Interestingly, ancestral sequence reconstruction exhibits a stepwise mutation pattern (figure 4) that starts around the time when the tested bat species first evolved from a common ancestor around 58 mya [21]. The ancestor of all bats maintains most of the original Laurasiatheria gene form (with only two mutations) after divergence with non-bat Laurasiatheria species; the ancestor of Hipposideridae, Rhinolophidae, and Megadermatidae (origin around 52 mya) has 3 mutations; the ancestor of Hipposideridae and Rhinolophidae (origin around 39 mya) has 4 mutations; the ancestor of Pteropodidae (origin around 23 mya) has 7 mutations; and the ancestor of the recently emerged *Pteropus* bats (around 3 mya) [25] have 13 mutations, hence showing a stepwise accumulation of mutations during bat *GULO* evolution.

## Discussion

As GULO is present in all major vertebrate lineages except some bats (most of these being New World species) [8], anthropoid primates [12], [26], guinea pigs [11], some passerine birds [27], and some fishes [5], such loss-of-function is neither related to broad phylogenetic affiliations nor to diet [28]. Some researchers have even proposed that the loss of Vc synthesis is associated with higher speciation rates because of higher mutation rates [29], which seems unlikely and which has not been tested formally.

Having successfully cloned bat *GULO* genes from 16 species, we carried out detailed evolutionary analyses. Our results show a range of forms of the *GULO* gene in bats. Combined with our earlier functional studies [14], we identify the following conditions: 1) pseudogenes that will have lost function, as seen in some *Pteropus* and hipposiderid species, 2) intact genes that functional studies showed loss of function in Vc synthesis (e.g. *Pteropus vampyrus*), 3) intact genes that maintain some ability to synthesize Vc (*Rousettus leschenaultii* and *Hipposdieros armiger*). We found that strong purifying selection has shaped non-*Pteropus* bat pseudogenes, suggesting these bats are in early stages of loss in their ability to synthesize Vc. In the family Hipposideridae some species possess pseudogenes that show only small changes from the intact and functional genes of their close relatives. Together with the evidence for puryifying selection our results suggest that Vc function has been lost recently in hipposiderid species showing pseudogenized *GULO*. Relaxed selection acting on *Pteropus* bat *GULO* suggests that bats in this genus lost the ability to synthesize Vc within the past 3 mya [25]. Thus we infer that pseudogenization of bat *GULO* evolved recently.

Ancestral reconstruction clearly shows a stepwise accumulating mutation pattern during bat *GULO* evolution. By mapping each mutation step with theorigination times of each clade (figure 4), we surprisingly found that the more ancient the species are, the less mutations they had accumulated; conversely, more recently evolved bats often accumulated many mutations, which supports our hypothesis that Vc synthesis involving *GULO* is gradually becoming less important in bats. The ancestral bats were therefore presumably able to biosynthesize Vc, and during evolution, *GULO* gene function is gradually becoming redundant.

In conclusion, our study shows that bats are beginning to lose their ability to biosynthesis vitamin C and some have lost this ability in no more than 3 mya. During gene degeneration, stepwise mutation patterns are evident and these are important mechanisms leading eventually to pseudogenization.

## Supporting Information

Table S1 Bat GULO genes with taxa information.
(XLS)

Table S2 Primers used for amplification of exon-3–exon-8 of bat *GULO* genes.
(DOC)
