# Supplementary material for: Recent Loss of Vitamin C Biosynthesis Ability in Bats
Source: PLoS One. 2011 Nov 1;6(11):e27114. doi: 10.1371/journal.pone.0027114 (PMC3206078; doi:10.1371/journal.pone.0027114)
Supplement: Table S2 — Primers used for amplification of exon-3–exon-8 of bat GULO genes. (DOC) [file pone.0027114.s002.doc]

Supplementary table S2. Primers used for amplification of exon-3 – exon-8 of bat *GULO* genes.

| Primer | Primer sequence | at (°C)^1^ | Product length |
| --- | --- | --- | --- |
| **exon-3** |  |  |  |
| 3F1 | 5'-TCA CTT CCT GGC CCT TCT CTC CGG CTC T-3' |  |  |
| 3R1 | 5'- GTG GAC ATC CTC ACC ATG GCC ACA GGG-3' | 61.9°C | ~530-bp |
| **exon-4** |  |  |  |
| 4F1 | 5'-GCT TGA GCT AAC CAC AGG CTG AGA CA-3' |  |  |
| 4R1 | 5'-CCT AAA AAT GGG AGA AGT GGC TCT TAC-3' | 59.7°C | ~220-bp |
| **exon-5** |  |  |  |
| 5F1 | 5'-AGA TGA ATC TAC TAT TCT GCT CTG AGG-3' |  |  |
| 5R1 | 5'-GCC CTT TTC CAC AAG GTG GCC AGG AGG CTC-3' | 59.7°C | ~490-bp |
| 5F2 | 5'-TGT CCT GCT TTG CCC AGC TTG-3' |  |  |
| 5R2 | 5'-GCA GTC AGC AGA GTG CTG CAA-3' | 57.5°C | ~150-bp |
| **exon-6** |  |  |  |
| 6F1 | 5'-CAG AGA GGT GAG GGC TTC ACC TGA AGG GTG-3' |  |  |
| 6R1 | 5'-CTC TGA GCT TCA CTG ACC TGC TCA-3' | 57.1°C | ~470-bp |
| 6R2 | 5'-AGA GAC CAG TAC CTC TCT CAG G-3' | 57.5°C | ~220-bp |
| **exon-7** |  |  |  |
| 7F1 | 5'-GGG AAG TCA GCG TGG AAG TGG ACC ATT TT-3' |  |  |
| 7R1 | 5'-CCC TTG ACC TCA CCC CTA CAC ATC CCT ACC-3' | 57.1°C | ~540-bp |
| 7F2 | 5'-CCG AGG TCA GGT TCT TGA CA-3' |  |  |
| 7R2 | 5'-GAT GAT AG/AC AAG CAT TGG CTG C-3' | 57.5°C | ~210-bp |
| **exon-8** |  |  |  |
| 8F1 | 5'-GGG AAC TGA CTG TGC CAC CAA GGA GTG A-3' |  |  |
| 8R1 | 5'-CAG ACA TGG GGC CCG AGC ATG AGC ACA GA-3' | 57.1°C | ~480-bp |
| 8R2 | 5'-GAC CAC ATC CTC AA/GA AGC ATA-3' | 57.5°C | ~320-bp |

^1^ annealing temperature
